# PPARγ as a sensor of lipase activity and a target for the lipase inhibitor orlistat

**DOI:** 10.1186/1476-511X-12-48

**Published:** 2013-04-08

**Authors:** Harry Martin, Tony K McGhie, Kerry Bentley-Hewitt, John Christeller

**Affiliations:** 1 Food Innovation, the New Zealand Institute for Plant & Food Research Ltd, Private Bag 11 600, Palmerston North 4442, New Zealand

**Keywords:** PPARγ, Orlistat, Xenical, Lipstatin, Lipase, Caco-2

## Abstract

A PPARγ fluorescence polarization (FP) assay was used to measure the release of fatty acid products from triglyceride emulsions during digestion with pancreatic and yeast lipases in a real-time, homogenous assay. Using the same FP assay we show the anti-obesity drug Orlistat is a PPARγ ligand with an IC_50_ of 2.84 ± 0.16 μM. Analytical Mass Spectrometry confirms that Orlistat does not bind covalently to PPARγ. The PPARγ FP assay is shown to be a simple method for measuring real-time lipase activity using a number of triglyceride substrates including olive oil and grape seed oil emulsions. Incubation of Orlistat with the human intestinal epithelial cell line Caco-2, at concentrations of 1 - 100 μM, leads to induction of genes regulated by PPARγ. At 100 μM Orlistat, transcription of β-defensin 1 (hDB1) & Adipose Differentiation Related Protein (ADRP) increase by up to 2.6 fold and 6.8 fold, respectively. Although at 1 μM and 100 μM Orlistat did not significantly increase defensin protein synthesis, at 10 μM Orlistat induced a 1.5 fold increase in hDB1 protein secretion in the human colonic adenocarcinoma cell line HT-29. Thus Orlistat is similar to the anti-diabetic drug Rosiglitazone in its ability to induce defensin gene expression. The antimicrobial peptide β-defensin 1 protects against pathogenic micro-organisms in the gut and PPARγ suppresses inflammatory gene expression. These may be beneficial side effects of Orlistat consumption on gut epithelial cells.

## Background

The PPARγ agonist and anti-diabetic drug Rosiglitazone is known to induce expression of the human β-defensin 1 (hBD1) gene in human intestinal epithelial Caco-2 cells [1]. Defensins are cationic peptides expressed in phagocytic and epithelial cells that lyse micro-organisms by forming pores in their membranes. Deficiency of defensin expression is associated with colonisation of the gut with *Candida albicans*[2] and also with Crohn’s disease [3]. Rosiglitazone has been used in the treatment of ulcerative colitis [4] but it has been associated with adverse cardiovascular effects in the treatment of type 2 diabetes [5].

The lipase inhibiting drug Orlistat is marketed by Roche under the trade name Xenical. It has a worldwide distribution and is used as an oral treatment for obesity [6]. Orlistat is also known as tetrahydrolipstatin and is a modified form of the streptomyces-derived lipase inhibitor lipstatin [7]. Orlistat is very hydrophobic: (logP 7.6 - 8.1) and binds covalently to the active site serine of pancreatic lipase [8]. Only trace amounts of Orlistat are absorbed systemically and the drug remains almost entirely in the gut lumen [9]. However, Orlistat penetrates cell membranes sufficiently to have intracellular effects [10,11]. Liposarcoma cell growth is inhibited at a concentration of 20 μM via inhibition of the intracellular enzyme, fatty acid synthase [12]. For this reason, Orlistat’s anti-cancer properties are being explored in a number of cancerous tissues including colorectal [13], prostate [14] and leukemic cells [15]. The extreme hydrophobicity of Orlistat led us to speculate that it might also behave as a PPARγ agonist in the gut epithelium where, due to its retention in the gut lumen, the local Orlistat concentration is high.

PPARγ binding activity was assayed by fluorescence polarization (FP) and mass spectrometry. Steady state mRNA levels of some PPARγ regulated genes in human Caco-2 epithelial cells were determined, including beta defensin-1, (hBD1), adipose differentiation related protein (ADRP) and PPARγ itself. In addition, due to the ability of PPARγ to bind a wide variety of fatty acids [16] we explored the utility of the PPARγ FP assay as a real-time assay for lipase activity.

## Results

### Real time lipase assay using PPARγ fluorescence polarization method

**Figure 1:**
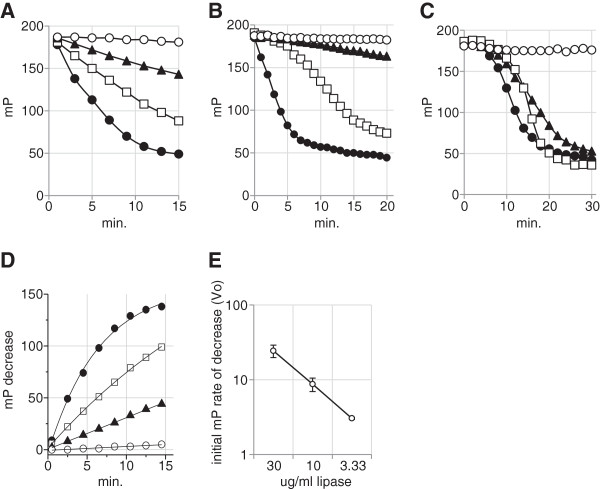
**Time course of triglyceride emulsion digestion measured by a PPARγ FP assay.A**: Porcine pancreatic lipase digestion of 1.5 mg/mL triolein. Lipase concentration: 30 μg/mL (●), (□), ▲ 3.3 μg/mL, no enzyme (○). **B**: Candida rugosa lipase (10 μg/mL) digestion of grape seed oil emulsion. Substrate concentration: 1.5 mg/mL (●), 0. 15 mg/mL (□), (▲) 0.015 mg/mL, no substrate (○). **C**: Digestion of three different substrates each at 0.15 mg/ml with 10 μg/ml *Candida rugosa* lipase: grape seed oil (●), olive oil (□) and triolein (▲). **D**: Data from Figure 1A plotted as increasing mP change and fitted for initial rate using MonoMolecular Curve fit to determine initial velocity. **E**: Initial velocities of lipase digestion reactions from Figure 1A/1D.

*Candida rugosa* lipase and porcine pancreatic lipase were incubated at concentrations ranging from 30 μg/mL to 3.3 μg/mL with various triglyceride emulsions in the presence of the FP reagents and polarization readings were taken at 1–2 minute intervals for up to 30 minutes. The PPARγ binding products released from the triglyceride emulsions were detected by the FP assay. Figure 1A shows the release of PPARγ binding products from digestion of varying concentrations of grape seed oil emulsion with *Candida rugosa* lipase. Figure 1B shows the release of PPARγ binding products from triolein using different concentrations of porcine pancreatic lipase. Figure 1C shows that release of PPARγ binding ligands during the digestion of emulsions of three different substrates viz. grape seed oil, triolein, and olive oil. To confirm the utility of the FP assay as a lipase assay, the initial velocities (V_o_) of the enzyme rates (from Figure 1A) at the three different concentrations were estimated and shown to be linear over a 9-fold dilution range (Figures 1D and 1E). These experiments have been repeated at least three times and the results shown are representative of the assay data which are highly reproducible. Because the FP assay is carried out in a 20 μl volume in a 384 well microplate, running replicates is simple and inexpensive.

### Measurement of Orlistat binding to PPARγ by Fluorescence Polarization

**Figure 2:**
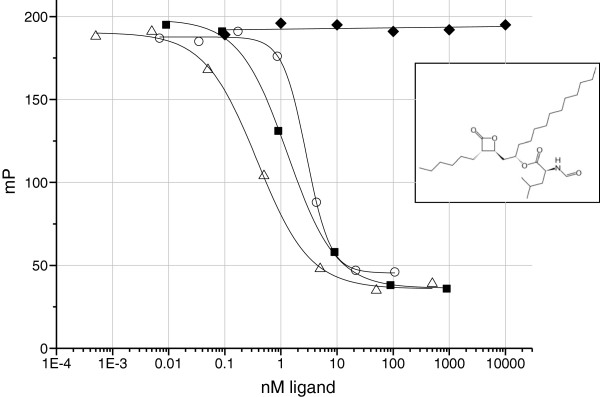
**Dose response curves of Orlistat (○), Troglitazone (■) and Rosiglitazone (△) and 5-aminosalicylic acid (**♦**) in a PPARγ FP assay.** The structure of Orlistat is shown inset.

Although lipase activity is readily traced by the release of fatty acids from the triglyceride substrate, the use of PPARγ FP assay as a lipase assay has the limitation that lipase inhibitors will tend to bind directly to the PPARγ due to their hydrophobic nature. Figure 2 shows that Orlistat is a PPARγ ligand with an IC_50_ of 2.84 μM, ±0.16. By comparison, the PPARγ agonists Troglitazone and Rosiglitazone are shown with IC_50_ values of 1.27 μM ±0.08 and 0.37 μM ±0.04 respectively.

### Orlistat does not modify PPARγ covalently

**Figure 3:**
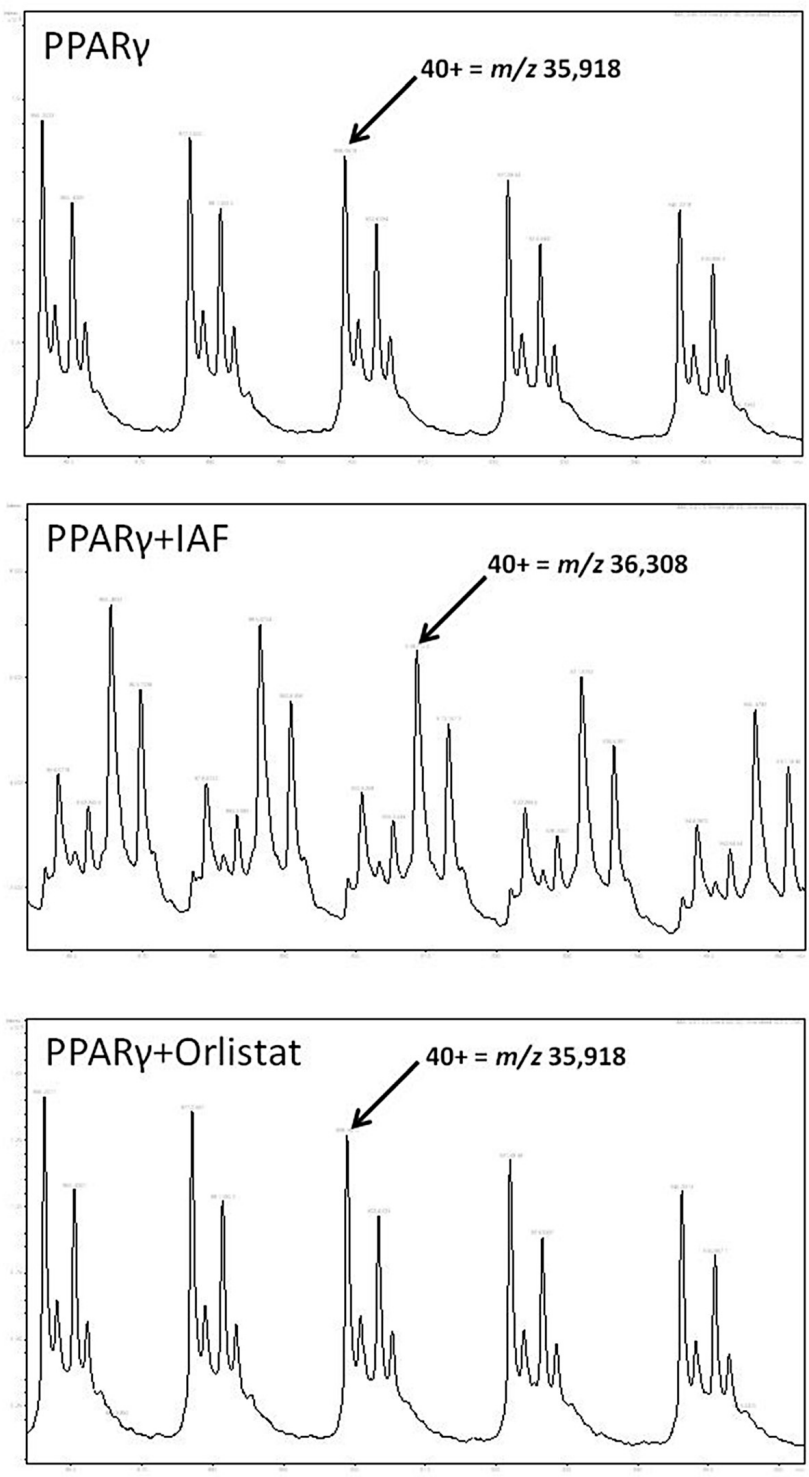
**The molecular weights of PPARγ and PPARγ conjugates were measured by ESI-MS.** The software package DataAnalysis (Bruker, Bremen, Germany) was used to average sample spectra and molecular weights calculated following charge deconvolution. The displayed mass spectra are of the molecular ion containing 40 positive charges and clearly show that the molecular weight of PPARγ increased when IAF is bound, indicating covalent binding. Whereas, there is no change in the molecular weight of PPARγ when Orlistat is bound, suggesting that the binding of PPARγ to Orlistat is by non-covalent mechanisms.

Orlistat (Figure 2) forms a covalent adduct with pancreatic lipase and contains 3 carbonyl groups. Several carbonyl containing fatty acids are known to bind covalently to the Cys285 in the ligand binding pocket of PPARγ [17]. For this reason we investigated the possibility of covalent modification of PPARγ by Orlistat by mass spectrometry. Orlistat was incubated in ammonium acetate buffer pH 7.4 with PPARγ for 1 hour at room temperature and then analysed by LCMS. The sulfhydryl-specific reagent iodo-acetamido fluorescein (IAF) was included to confirm that this procedure was able to detect covalent modification of the PPARγ. The molecular weight of the PPARγ was confirmed at 35,918 Da and is consistent with data provided by the supplier. When IAF is added the molecular weight of PPARγ increased to 36,308 Da, an increase of 390 Da, consistent with the addition of IAF to a sulfhydryl group on the PPARγ molecule. However, the molecular weight of PPARγ remained at 35,319 Da when Orlistat was added, suggesting that Orlistat does not form covalent bonds with PPARγ (Figure 3). In addition, two covalent PPARγ ligands, Dithio-bis (2-nitrobenzoic acid) and GW9662, were also confirmed bind to PPARγ irreversibly (data not shown).

### Orlistat induces hBD1, ADRP & PPARγ mRNA expression in Caco-2 cells

**Figure 4:**
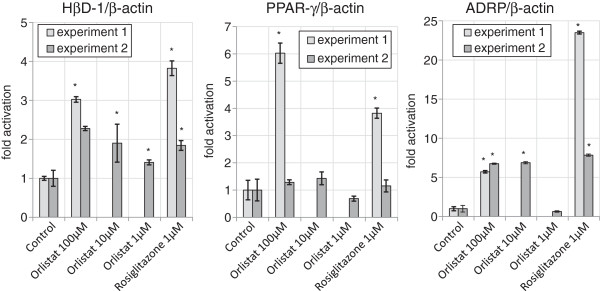
**Relative gene expression of hBD1, PPARγ and ADRP in Caco-2 cell following exposure to Orlistat, Rosiglitazone or DMSO control for 24 h.** Individual results were normalised to β-actin. Each bar represent mean ± SD (n=3). Significant differences between treatment and control are shown as *(p<0.05).

Treatment of Caco-2 human intestinal epithelial cells with the PPARγ activator Rosiglitazone is known to induce the induction and expression of the hBD1 gene and Adipose Differentiation Related Protein (ADRP). To determine whether Orlistat is also a PPARγ activator we treated Caco-2 cells with 100 μM Orlistat or with 1 μM Rosiglitazone for 24 hours before cells were harvested for analysis of gene induction. Following the initial observation that 100 μM Orlistat induced hBD1 gene expression, the experiment was repeated using Orlistat at 100, 10 and 1 μM. The results of both experiments are shown in Figure 4. In the first experiment gene transcription of hBD1, PPARγ, ADRP and were all significantly enhanced by treatment with 100 μM Orlistat by 3.0, 6.0 and 5.7 fold respectively and also by Rosiglitazone, the positive control. In the second experiment, significant increases in defensin gene transcription occurred at all three concentrations of Orlistat. Even at 1 μM Orlistat a 1.4 fold increase in defensin transcription was observed. In the second experiment, ADRP gene transcription was increased by around seven fold using Orlistat at 100 μM and 10 μM but no increase was observed at 1uM Orlistat. For reasons unknown, the PPARγ gene induction seen in the first experiment was not observed in the second experiment.

### Orlistat induces hBD1 protein expression in expression in HT-29 cells

**Figure 5:**
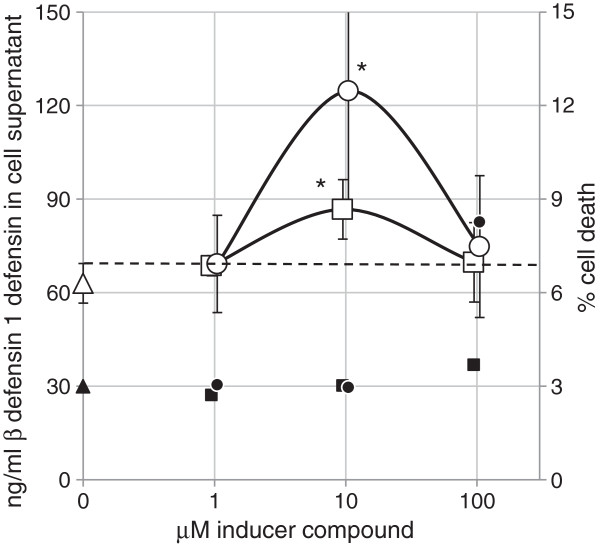
**hBD1 protein expression in HT29 cell supernatants and cell death following 24 h exposure to Orlistat, Rosiglitazone and DMSO control. Each datapoint represents mean ± SD (n=3).** Significant differences between treatment and control are shown as *(p<0.05). The dashed line indicates the upper value of the error bar for the DMSO control sample. hBD1 protein in DMSO control cell supernatant (Δ), hBD1 protein in Rosiglitazone treated cell supernatant (○),hBD1 protein in Orlistat treated cell supernatant (□). Tissue culture percent cell death at 24 h; DMSO control cells (▲), cell death in Rosiglitazone treated cells (●), cell death in Orlistat treated cells (**■**).

To establish whether the Orlistat and Rosiglitazone induced β defensin1 mRNA expression was also associated with an increase in β defensin1 protein synthesis, we first performed a sandwich ELISA on the tissue culture supernatants of Caco2 cells treated with Orlistat. We were unable to detect β defensin1 protein in the Caco2 supernatants although Caco2 are reported to synthesis β defensin1 [18]. The related cell line HT-29 (a human colonic adenocarcinoma) was then used as an alternative to Caco2 (also human colorectal adenocarcinoma). Orlistat & Rosiglitazone we tested at concentrations of 1, 10 and 100 μM for 24 h in culture. The supernatant was analysed by sandwich ELISA for hBD1 protein and the results are shown in Figure 5. Although the average defensin protein was increased at all concentrations of drug treatment, significant increases in defensin levels were only found at 10 μM Orlistat (~1.5 fold increase) & 10 μM Rosiglitazone (~2 fold increase).

## Discussion

The PPARγ FP assay provides a homogeneous and high-throughput method (Figure 1) for continuous monitoring of lipase reaction kinetics. The sensitivity of the assay is determined by the affinity of the fatty acid product for PPARγ ligand binding domain, which for many PPARγ ligands is in the low micromolar range. Since PPARγ is a non-selective binder of various fatty acids, the assay is will not distinguish different lipase products. The overlapping curves in Figure 1C may therefore reflect the formation of different products with different affinities for PPARγ. Triolein yields oleic acid as a lipase digestion product whereas around 70% of the fatty acid content of grape seed oil is linoleic acid. The wide range of fatty acids which are PPARγ ligands means that the assay can be applied to the analyses of many lipases & substrates. At high concentrations of substrate (Figure 1A, 1E), the rate of product formation is directly correlated with lipase concentration whereas at low substrate concentrations (Figure 1B, 1C) a distinct lag period is evident before product formation becomes detectable, giving the reaction curve a sigmoidal appearance. The reasons for the lag period are unclear at present and may reflect a combination of causes; the use of substrate at concentrations lower than the lipase K_m_; partitioning of the emulsion substrate and enzyme at low substrate concentrations; a low threshold of product formation may be required before the fluorescent ligand can be displaced from the PPARγ receptor in the FP reaction. Nonetheless, as Figure 1A shows, the FP assay is extremely simple and sensitive as a method detecting and for quantifying lipase in a sample. FP, as a ratiometric technique, is less sensitive to sample colour and opacity than direct fluorescence intensity methods. However, as the Orlistat data shows (Figure 2) the PPARγ FP procedure is unlikely to permit the analysis of lipase inhibitors since their lipophilicity will tend to make them PPARγ ligands. It is well known that cytosolic phospholipases initiate signal transduction pathways by releasing fatty acid signalling molecules from phospholipid membranes and that many of the phospholipase products are PPAR ligands. Thus, the *in vitro* generation of PPARγ ligands from lipase activity shown in Figure 1 is analogous to the situation in cells whereby phospholipase A_2_ activation results in the generation of PPARδ ligands and the subsequent expression of PPARδ dependent genes [19].

The possibility of covalent bond formation between Orlistat & PPARγ was investigated because Orlistat binds covalently to pancreatic and other lipases [8] and because keto-fatty acids are known to form a covalent bond with Cys285 in the ligand binding pocket of PPARγ [17]. The mass spectrometry data shown in Figure 3 clearly demonstrated that Orlistat, which contains 3 carbonyls [7], does not bind covalently to PPARγ since no adduct was detectable by mass spectrometry.

The induction of beta defensin-1, and ADRP by Orlistat (Figure 4) in Caco-2 cells along with the direct binding of Orlistat to PPARγ (Figure 2), is strong evidence that Orlistat is a PPARγ agonist since these genes are known to be induced by the PPARγ agonist drugs Troglitazone and Rosiglitazone [1,20]. In Figure 4, Orlistat was used at a concentration as high as 100 μM because this concentration in the gut lumen would be achieved by the standard daily dose of 0.36 g Orlistat [21] coupled with almost complete retention of the drug in the in the gastrointestinal tract [9]. Although Orlistat will induce cell death in human colonic adenocarcinoma HT29 cells at a concentration of 100 μM over 48 hours it does not induce cell death at 200 μM over a period of 24 hours [13] and it is far less toxic to normal cells than to cancerous cells [14,15]. Furthermore, we see no evidence of cell death in our Caco-2 culture, for example, by detecting lower levels of control GAPDH RNA. Although Rosiglitazone was slightly toxic to HT-29 cells at 100 μM (Figure 5) no evidence of toxicity was observed at 10 μM at which a two-fold increase in β-defensin 1 protein was observed. Peyrin-Biroulet et al. (2010) has shown that Rosiglitazone induced hBD1 mRNA. Our data now confirms that hBD1 protein is also induced by Rosiglitazone. Although the Orlistat induced defensin protein is only increased by 1.5 fold at 10 μM, this may result in a significant physiological effect in patients in terms of pathogen resistance. A future study of the faecal hBD1 protein content in Orlistat patients might confirm a PPARγ mediated physiological benefit of Orlistat.

Orlistat suppresses the onset of type 2 diabetes [22] due the reduced calorific intake, and concomitant weight loss of patients. As a consequence of Orlistat’s PPARγ agonist activity, there may be local insulin sensitising effects in the gut epithelium and additional physiological responses including increased hBD1 and PPARγ transcription.

PPARγ activation is enhanced by the actions of cytosolic fatty acid binding proteins which deliver sparingly soluble fatty acid ligands to the nucleus [23]. This raises the question of whether Orlistat also binds to intracellular fatty acid binding proteins in gut epithelial cells and what the consequences of that putative binding might be. Orlistat consumption is well known to cause steatorrhea due to the arrival of undigested fat in the colon. Although Orlistat is an irreversible inhibitor of pancreatic lipase, it is a reversible inhibitor of certain bacterial lipases and not an inhibitor of a staphylococcal extracellular lipase [24]. Some colonic digestion of fats is likely to occur through bacterially-derived lipases in patients using Orlistat. The fatty acids produced in these circumstances may induce PPARγ dependent gene transcription in the epithelial cells of the intestine in the same way that Orlistat affects Caco-2 cells. Thus, in addition to pro-apoptotic side effects of Orlistat in colorectal carcinoma cells [13], Orlistat may also induce anti-inflammatory genes in gut tissue mediated by its PPARγ agonist activity.

## Conclusions

The widely used lipase inhibitor drug Orlistat binds reversibly to PPARγ with an IC_50_ of 2.8 μM. Orlistat acts on gut epithelial Caco-2 cells as a PPARγ agonist and increases synthesis of human defensin β1 and ADRP mRNA.

## Methods

### Chemicals and materials

Rosiglitazone, Troglitazone, 5-amino salicylic acid, GW9662 (a covalent binding PPARγ antagonist [25]), Orlistat, triolein and iodo-acetamido-fluorescein were supplied by Sigma-Aldrich. Grape seed oil and olive oil were commercial brands from local supermarkets in Palmerston North, New Zealand. LC-MS grade acetonitrile was from Thermo Scientific, methanol (ChromAR) was from Mallinckrodt Chemicals, and ethanol (95%) was from LabServ.

### Fluorescence Polarization assay of PPARγ ligands

FP assays were performed on the Tecan Safire2 fluorescence microplate reader (Tecan, Austria) at 22°C, in a volume of 20 μL in Nunc 384 well, black, shallow microplates. The PPARγ (green) competitive binding assay (PolarScreen™) kit was supplied by Invitrogen Corporation, Carlsbad, CA, USA. For measurement of FP, excitation and emission wavelengths were set at 470 nm and 525 nm. Sigmoidal and exponential curve fitting and IC_50_ and V_o_ estimates were performed with Origin software (Origin-Lab, Northampton, MA, USA) using the logistic and MonoMolecular equations respectively*.* Data presented are representative of at least two independent experiments.

### Lipase assays

Porcine pancreatic lipase (L3126) and *Candida rugosa* lipase (L1754) was supplied by Sigma. Triolein, grape seed oil & olive oil were used as triglyceride substrates for lipase assays. Lipase substrate was prepared according to the method of Nilsson-Ehle [26]. Briefly, substrate emulsions were prepared by mixing 240 mg triglyceride substrate and 40 mg whey protein (WP1485) in 10 g glycerol. 200 μL of this stock solution was added to 800 μL 50 mM Hepes buffer pH7.5 which was sonicated for six periods of 5 sec at 6 watts for each mL of assay mix. For FP assays, the substrate emulsions were diluted in Invitrogen FP PPARγ assay buffer to a final concentration of 0.15 mg/mL triglyceride and digested with lipase for 30 minutes at 22°C in volumes of 20 μL. During the digestion period, FP readings were taken at 1 or 2 minute intervals.

### LC-QTOF-HRMS

The LC-MS system was composed of a Dionex Ultimate® 3000 Rapid Separation LC system and a micrOTOF QII mass spectrometer (Bruker Daltonics, Bremen, Germany) and was operating in positive mode with an electrospray ionization source. The LC system contained a SRD-3400 solvent rack/degasser, HPR-3400RS binary pump, WPS-3000RS thermostated autosampler, and a TCC-3000RS thermostated column compartment. The analytical column was a Zorbax™ SB-C18 2.1 × 100 mm, 1.8 μm (Agilent, Melbourne, Australia) maintained at 50°C and operated in gradient mode. Solvents were A = 0.5%formic acid, and B = 100%acetonitrile at a flow of 400 μL/min. The gradient was: 70%A, 30%B, 0–0.5 min; linear gradient to 45%A, 55%B, 0.5-25 min; linear gradient to 2%A, 98%B, 25–45 min; composition held at 2%A, 98%B, 45–50 min; linear gradient to 70%A, 30%B, 50–50.2 min; to return to the initial conditions before another sample injection at 54 min. The injection volume for samples and standards was 2 μL. The micrOTOF QII source parameters were: temperature 200°C; drying N2 flow 8 L/min; nebulizer N2 4.0 bar, endplate offset -500 V, capillary voltage −4000 V; mass range 100–1500 Da, acquired at 2 scans/s. Post-acquisition internal mass calibration used sodium formate clusters with the sodium formate delivered by a syringe pump at the start of each chromatographic analysis.

### Tissue culture

Caco-2 cells and HT-29 cells were cultured in DMEM supplemented with 5%Foetal bovine serum, glutamine & antibiotics at 37°C, 5%CO_2_. Test compounds in DMSO were tested in triplicate by addition of 1 μl of test compound to 1 ml cell culture for 24 h.

#### Real-Time RT-PCR

Total RNA was extracted by the use of High Pure RNA Isolation Kit and reverse transcription performed using Transcriptor First Strand cDNA Synthesis kit with oligo-dT primed reactions, both according to manufacturer’s instructions (Roche Diagnostics, New Zealand). Primers and primer-dual hybridisation probe combinations (Roche Diagnostics, Germany-Table 1) were designed online using the Universal probe library system assay design centre (Roche Applied Science). The RT-qPCR assay was performed using the Lightcycler® 480 system (Roche Diagnostics, Germany) with three reactions (technical replicates) for each sample. Real-time PCR parameters are as follows: 10 minutes (0:10:00) pre-incubation at 95°C, 40 cycles of amplification from 95°C (0:00:10), to 58°C (0:00:20), to 72°C (0:00:01), followed by cooling at 40°C (0:00:10). Results were relative to the expression of glyceraldehyde-3-phosphate dehydrogenase and β-actin, however the latter produced most consistent expression between samples and was used as the reference gene to calculate final relative expression.

**Table 1:** qPCR oligonucleotides and RT-qPCR efficiencies for Caco-2 cells

**Gene name /(genebank accession no.)**	**Primer and probe sequences**
GAPDH: Glyceraldehyde-3-phosphate dehydrogenase (NM_002046.3)	F: AGCCACATCGCTCAGACAC
	R: GCCCAATACGACCAAATCC
ACTB, β-actin (NM_005345.4)	F: GGAGTCCTACGCCTTCAACA
	R: CCAGCACCTTCTTCTTGTCG
hBD1, Human β defensin 1 (X92744.1)	F: TGTCTGAGATGGCCTCAG GT
	R: GGGCAGGCAGAATAGAGACA
ADRP, Adipose Differentiation Related Protein (NM_001122.2)	F: TCAGCTCCATTCTACTGTTCACC
	R: CCTGAATTTTCTGATTGGCACT
PPARγ variant 1 (NM_138712.3)	F: GACAGGAAAGACAACAGACAAATC
	R: GGGGTGATGTGTTTGAACTTG

### Statistical analysis

Quantitative RT-PCR results were analysed using inbuilt relative quantification software (Light –Cycler 480 software version 1.0), using the standard curve for both target and reference (ACTB) gene and the software then determined the target to reference ratio.

### β-defensin ELISA

24 h tissue culture supernatants were analysed in triplicate for human-β defensin 1 using a commercial sandwich ELISA kit supplied by Genway, San Diego.

## Competing interests

None of the authors have a financial conflict of interest in regards to the materials included in this paper.

## Authors’ contributions

Orlistat, Lipases and triglyceride substrates were supplied & prepared by JC. Fluorescence Polarization assays were performed by HM. Mass spectrometry was carried out by TMcG. RT-PCR & defensin ELISAs were performed by KBH. All authors read and approved the final manuscript.
